# A Digital Intervention Addressing Alcohol Use Problems (the “Daybreak” Program): Quasi-Experimental Randomized Controlled Trial

**DOI:** 10.2196/14967

**Published:** 2019-09-04

**Authors:** Robert J Tait, Raquel Paz Castro, Jessica Jane Louise Kirkman, Jamie Christopher Moore, Michael P Schaub

**Affiliations:** 1 National Drug Research Institute Faculty of Health Sciences Curtin University Perth Australia; 2 Swiss Research Institute for Public Health and Addiction University of Zurich Zurich Switzerland; 3 Hello Sunday Morning Sydney Australia

**Keywords:** alcohol consumption, internet, digital health, intervention study, social marketing, health promotion

## Abstract

**Background:**

Alcohol use is prevalent in many societies and has major adverse impacts on health, but the availability of effective interventions limits treatment options for those who want assistance in changing their patterns of alcohol use.

**Objective:**

This study evaluated the new *Daybreak* program, which is accessible via mobile app and desktop and was developed by *Hello Sunday Morning* to support high-risk drinking individuals looking to change their relationship with alcohol. In particular, we compared the effect of adding online coaching via real-time chat messages (intervention group) to an otherwise self-guided program (control group).

**Methods:**

We designed the intervention as a randomized control trial, but as some people (n=48; 11.9%) in the control group were able to use the online coaching, the main analysis comprised all participants. We collected online surveys at one-month and three-months follow-up. The primary outcome was change in alcohol risk (measured with the alcohol use disorders identification test–consumption [AUDIT–C] score), but other outcomes included the number of standard drinks per week, alcohol-related days out of role, psychological distress (Kessler-10), and quality of life (EUROHIS-QOL). Markers of engagement with the program included posts to the site and comments on the posts of others. The primary analysis used Weighted Generalized Estimating Equations.

**Results:**

We recruited 398 people to the intervention group (50.2%) and 395 people to the control group (49.8%). Most were female (71%) and the mean age was 40.1 years. Most participants were classified as probably dependent (550, 69%) on the AUDIT–10, with 243 (31%) classified with hazardous or harmful consumption. We followed up with 334 (42.1%) participants at one month and 293 (36.9%) at three months. By three months there were significant improvements in AUDIT–C scores (down from mean 9.1 [SD 1.9] to 5.8 [SD 3.1]), alcohol consumed per week (down from mean 37.1 [SD 28.3] to mean 17.5 [SD 18.9]), days out of role (down from mean 1.6 [SD 3.6] to 0.5 [SD 1.6]), quality of life (up from 3.2 [SD 0.7] to 3.6 [SD 0.7]) and reduced distress (down from 24.8 [SD 7.0] to 19.0 [SD 6.6]). Accessing online coaching was not associated with improved outcomes, but engagement with the program (eg, posts and comments on the posts of others) were significantly associated with improvements (eg, in AUDIT–C, alcohol use and EUROHIS-QOL). Reduced alcohol use was found for both probably dependent (estimated marginal mean of 40.8 to 20.1 drinks) and hazardous or harmful alcohol users (estimated marginal mean of 22.9 to 11.9 drinks).

**Conclusions:**

Clinically significant reductions in alcohol use were found, as well as reduced alcohol risk (AUDIT–C) and days out of role. Importantly, improved alcohol-related outcomes were found for both hazardous or harmful and probably dependent drinkers. Since October 2016, *Daybreak* has reached more than 50,000 participants. Therefore, there is the potential for the program to have an impact on alcohol-related problems at a population health level, importantly including an effect on probably dependent drinkers.

**Trial Registration:**

Australian New Zealand Clinical Trials Registry: ACTRN12618000010291; https://www.anzctr.org.au/Trial/Registration/TrialReview.aspx?id=373110

**International Registered Report Identifier (IRRID):**

RR2-10.2196/9982

## Introduction

The use of alcohol is one of the leading causes of disease burden and deaths globally, and among those aged 15-49 years it is the leading risk factor for deaths for both males and females [1]. While there are increasing doubts that alcohol use confers any health benefits [2], national guidelines tend to be framed in terms of reducing the risks to health from alcohol use [3,4]. Despite these guidelines, many people still consume alcohol in a manner that increases their risk of adverse consequences: in Australia, about 38% of those aged 12 years or older exceeded either the single occasion consumption guideline or the regular use guideline in 2016 [5]. Further, the current availability of treatment resources is estimated to only fulfil 26%-48% of the needs for treatment required [6]. In the United States, about 24.5% of those aged 12 years or older are classified as having binged in the last 12 months, about 6.1% have engaged in heavy alcohol use and about 5.1% have an alcohol use disorder [7]. Yet, even among those with an alcohol use disorder, less than 10% are likely to have received treatment in the last year [8].

In order to provide interventions, particularly for those with at-risk patterns of alcohol use (as opposed to those with alcohol use disorders, such as alcohol dependence), opportunistic screening and brief interventions have been developed [9,10]. Subsequently, these face to face interventions have been adapted for Internet delivery, with the intensity varying from personalized feedback to multistage interventions [11,12]. An early meta-analysis found that compared with single session interventions (Hedges’ *g*=.27), extended self-help interventions (Hedges’ *g*=.61) offered significantly greater reductions in alcohol use [11] but that the addition of guidance did not significantly improve outcomes compared with unguided interventions [11]. In contrast, a recent individual patient meta-analysis based on 14,198 participants found that guided interventions resulted in greater reductions in alcohol use than fully automated interventions [13]. Further, no difference in outcomes was found between those with different drinking profiles (described as regular at-risk drinking, heavy drinking or binge drinking.)

Even where interventions have been shown to be efficacious, there are few publicly available websites, with a recent review identifying 72 trials of alcohol interventions but only eight (11%) with functioning websites [14]. Further, the importance of interventions reaching the whole of the target population has been emphasized if public health impacts are to be realized [15]. To this end, *Hello Sunday Morning* took a different approach to conventional web-based interventions and provided both a social media–based, health promotion movement combined with an online environment that incorporated blogging, social networking, peer support and interventions (eg, behavioral experiments) [16].

*Hello Sunday Morning*, established in 2010, asked participants to set a public alcohol consumption goal (eg, abstinence or reduction) for a set period of time (eg, three, six or twelve months [16,17]). Previous reports have provided descriptive analysis of participants [16], and an observational study reported a significant improvement in scores on the alcohol use disorders identification test (AUDIT) [18], down from a mean of 20.3 (SD 6.7) at baseline to a mean of 8.9 (SD 8.9) at seven months [19]. In August 2018, the original version of *Hello Sunday Morning* closed, having registered more than 100,000 people, and was superseded by the *Daybreak* program that was accessible via mobile app and desktop.

As with the legacy program, the *Daybreak* program aimed to support individuals in changing their relationship with alcohol via access to peer support, self-guided experiments, and individualized health coaching. *Daybreak* was developed in response to the growing number of probably dependent drinkers engaging with *Hello Sunday Morning*, to ensure that these members had the level of clinical support they required (eg, access to health coaches, psychologists and appropriate levels of peer moderation) that the previous version of the program could not provide.

The objectives of the current study were: (1) to assess the effectiveness of the new *Daybreak* program; and (2) to evaluate the effect of adding a professional, clinical component (termed online coaching) to the program, whereby members had access to counsellors or psychologists via smartphone chat. We hypothesized that those receiving coaching would show greater improvements in alcohol-related measures than those who did not receive coaching. As additional objectives, we evaluated whether engagement with the program improved outcome measures. Further, given the need to provide resources for both those with at-risk alcohol use and those with more severe problems (probable dependence), outcomes were compared for these two groups. *Daybreak* is designed as a standalone intervention.

## Methods

### Design

The study was planned as a randomized control trial to compare the *Daybreak* program plus online coaching via real-time chat messages (Intervention group) and the *Daybreak* program without online coaching (Control group) at one, three and six months. However, due to a programming error, some (n=48; 11.9%) people in the Control group were able to access online coaching and we could not tell if other members of the Control group found they could access the online coaching but did not opt to use it. Therefore, with the approval of the human research ethics committee, the trial was ended after the last person to consent reached their three-month follow-up rather than the planned six-month period.

### Participants and Randomization

We invited new registrants to *Daybreak* to join the study (see Multimedia Appendix 1). To be eligible, participants had to be 18 years or older, a resident in Australia, had to provide a valid email address and had to have access to the Internet. However, we excluded those who reported a history of treatment for cardiovascular disease, as this was likely to confer additional health risks during alcohol withdrawal [20]. Participants had to exceed the at-risk threshold (>7) on the 10-item AUDIT [18]. Scores on the AUDIT between 8-19 indicate a pattern of drinking that is likely to fulfil the International Classification of Diseases criteria for hazardous or harmful drinking, while higher scores equate to alcohol dependence [21], but the AUDIT is a screening tool and not a diagnostic indicator. Finally, as part of our duty of care, we assessed suicide risk with the P4 suicidality screening survey [22]. Those participants classified as above minimal risk were still eligible for the study, but they were also provided with the contact details for Lifeline (a 24-hour support service). Those people classified in the highest AUDIT group (≥20: probable dependence) had it recommended to them to speak to their general practitioner or other health professional prior to reducing their use of alcohol in order to minimize potentially serious complications during alcohol withdrawal. The study planned to use a simple, fully randomized allocation (eg, no blocking). Participants were blind to their condition and follow-up data were collected online by an automated survey and hence blind to condition. Those not meeting the eligibility criteria could still access *Daybreak* but were not part of the study cohort. The processes of screening, enrolment and randomization were fully automated.

### Procedure

The *Daybreak* program is available from the Google and Apple App Stores or https://www.daybreakprogram.org/, but it can also be accessed from sites such as healthdirect.gov or other health directories. Alternatively, visitors to the *Hello Sunday Morning* website (www.hellosundaymorning.org) are directed to the program. *Daybreak* is a self-guided program that can be accessed as frequently as required by the participant, and access is controlled via username (email address) and password. Those in the Intervention group also had access to an online health coach between 7:00 and 19:00 on weekdays, but as noted above, some of the Control group also accessed coaching. We commenced recruitment in February 2018 and closed it in November 2018. The *Daybreak* version used throughout the study was version 1.5.8, build 90. Since late 2016, *Daybreak* has reached more than 50,000 participants.

We emailed and sent a text to participants with a link to the relevant follow-up survey after one month and after three months. If required, we sent a reminder text message one week later. If there was no response, a research assistant who was blind to study group allocation telephoned the participant to ask them to complete the follow-up, with a maximum of three telephone reminders calls allowed to be made. At each follow-up, participants were eligible to enter a draw to win an Ipad2. The study received the required institutional ethics approval (Curtin University 2017-0855), and the trial procedure was registered (ACTRN12618000010291). In compliance with Australian ethical guidelines, as all participant were screened with at risk alcohol use, we used an active rather than placebo control group [23].

### Sample Size

The sample size estimation was calculated for the planned outcomes at six months. For online alcohol interventions, typical effect size values are in the range of Cohen *d*=0.3-0.4 at 6 months [11,12,24]. We were not aware of previous investigations involving social networks as a means of reducing alcohol use; however, investigations with other behaviors (eg, diet, physical activity) typically report small but not significant changes [25]. Therefore, we based our sample calculation on a small effect (Cohen *f=*.10; equivalent to Cohen *d*=0.2) and assumed that the repeated measures would be correlated at *r*=0.5. To achieve a power of 0.80 with an alpha *P*<.05 would require 60 people per group, however, given the clinical interest in the effectiveness of brief alcohol interventions for those with more entrenched problems (probable dependence), and potential gender differences, we aimed to recruit 60 people to the smallest cell (sex by AUDIT risk level by study group). To achieve this, we projected recruitment of 300 participants in each group (600 in total), and assuming that 35% would be lost to follow-up, we targeted 467 per group (N=934).

### Outcome Measures

Participants were screened with the 10-item AUDIT, which has been validated in Australia [18] and elsewhere. Scores range from 0-40 (0-7=low risk, 8-19=hazardous or harmful alcohol use, 20-40=probable dependence), and outcomes were assessed as change in the scores for the first three items (termed the AUDIT–C). Prior research has shown that the AUDIT–C can predict clinical outcomes at 12 months [26]. We also assessed secondary outcomes and other measures, which are listed in Textbox 1.

**Secondary outcomes and other measures assessed in the study.**

Secondary Outcomes:

- Self-reported alcohol consumption in standard drinks (10 g alcohol) collected via a 7-day drinking diary [27,28]. Australian guidelines recommend no more than two standard drinks per day in the general adult population [3].
- Mental distress was assessed with the Kessler’s K-10 [29]. The K-10 scores have a range of 10-50. We interpreted values of 20-24 as showing mild distress, 25-29 as moderate distress and ≥30 as severe mental health distress [30].
- We used Kessler’s Days out of role [31] to determine the number of days either wholly or partially out of role due to alcohol consumption during the last 30 days. Research shows that people with an alcohol disorder have significantly more days either wholly or partially out of role than those without a disorder [31]. From national Australian data, for those with alcohol dependence, the mean number of days out of role is 3.8 [32].
- Quality of life was assessed with the eight item EUROHIS-QOL (also known as the World Health Organization QOL-8). This has been recommended for use in alcohol and other drug treatment services [33] and has been validated in Australia [34], with a depressed sample having a mean score of 2.71 (SD 0.69) versus a non-depressed group with a mean of 3.30 (SD 0.64).

Other Measures:

- The use of health services was quantified with a checklist of health professionals seen in the last eight weeks. This was adapted from a preexisting checklist [35] by the addition of alcohol or other drug treatment services and alcohol pharmacotherapy.
- A four-point rating item (very bad to very good) from the Pittsburgh Sleep Quality Index was used assess to sleep quality in the last four weeks [36].
- We used the Godin Leisure-Time Exercise survey to estimate total exercise in the last seven days [37]. This allows the metabolic equivalents (METs) from different types of exercise to be combined, with one MET defined as the energy used while sitting at rest [38].
- We quantified adverse events arising from alcohol use, only at baseline, using the CORE survey [39]. As this was originally developed for college populations, two items were modified (ie, “missed a class” changed to “missed a class or work” and “been in trouble with police, resident hall, or other college authorities” changed to “been in trouble with police or other authorities”). Further, as the current study focused on alcohol, the reference to drug use (lead-in statement and from one item “Thought I might have a drinking or other drug problem”) was deleted. For United States college students, more than 30% reported driving under the influence, and between 1/5 and 1/3 report being in an argument or fight[39].

Engagement with the *Daybreak* program was recorded as: (1) engagement with coaching (defined as at least one message sent by the participant to the coach); (2) The number of experiments completed; (3) number of blog shares (posts on their own blog); and (4) number of blog comments (posts on another person’s blog).

### Content of the Interventions

#### Overview

The rationale behind the *Daybreak* program is to help people change their relationship with alcohol. This is facilitated by encouraging participants to establish a goal (eg, abstinence, reduced use), to reflect on their mood, and also to give and receive peer support. Four mechanisms were used to help achieve behavior change, which included weekly check-ins, peer support, behavioral experiments and health coaching.

#### Weekly Check-Ins

The *Daybreak* program includes self-reported questionnaires to encourage participants to undertake self-reflection to explore their intrinsic motivators for change.

#### Peer Support

The *Daybreak* program enables participants to connect with other users of the program through a blog function. Internal data showed that 45% of shares on the blog received five or more comments in less than 60 minutes. In addition to supporting others going through the process of becoming a nonconsumer of alcohol, the narrative process enables individuals to construct new self-identities, with transitions in the narratives often noted [40].

#### Behavioral Experiments

Two components of the *Daybreak* program are self-guided experiments and associated learnings. The experiments draw on a range of theoretical perspectives (eg, cognitive behavioral therapy, acceptance and commitment therapy) and cover five areas (mindfulness, connectedness, resilience, situational strategies and health). For example, a mindfulness experiment might guide participants to be in the moment during a period of craving. There are also some experiments that take a broader perspective to help participants with their general health (eg, fitness routines, healthy eating, sleep hygiene).

#### Health Coaching

The online health coaching was the critical difference between the Intervention Group and Control group. As previously described [41], all the health coaches fulfilled the relevant guidelines for low intensity mental health providers [42], and some coaches were registered general and clinical psychologists. In-house training was provided to develop skills and knowledge on delivering online services (eg, ethical considerations, forming connections online, coaching procedures, risk management, and platform specific training). Novice coaches received supervision, with further periodic feedback from a senior health coach.

The role of the online coaches was to partner with participants in order to assist them with goal setting and assist them in reaching their goals. All coaching interactions occurred through real-time chat messages on a secure platform. The coaches tailored support to the individual’s requirements and drew on a range of techniques (eg, cognitive behavioral therapy, motivational interviewing and acceptance and commitment therapy techniques) as appropriate. *Daybreak* has a written Risk Management Protocol covering all members, not just the research cohort, to automatically detect trigger words and alert the clinical team [41]. In addition, forum posts were monitored and the clinical team responded to alerts from other participants.

### Analysis

Descriptive data and analyses (eg, *t* tests, Mann-Whitney *U* tests, χ^2^ tests) were provided for each group. However, due to concerns over the randomization process, the main repeated measures analysis was for all participants rather than by randomized groups. In addition to the overall results, Multimedia Appendix 2 provides online descriptive data at 1-month and 3-months follow-up by randomized group for each outcome measure.

We used Weighted Generalized Estimating Equation (WGEE) analyses to investigate longitudinal changes in primary and secondary outcomes over the study period. WGEE is a repeated measures regression model that takes into account the correlation of repeated measures within each subject [43]. In contrast to repeated measures analysis of variance (ANOVA), WGEE has minimal assumptions about time dependence and uses all available longitudinal data, irrespective of single missing values at follow-up. WGEE is also more robust than unweighted generalized estimating equation, when the assumption of missing completely at random (MCAR) is violated. To control for attrition at 1-month and 3-months follow-up, we estimated weights as suggested by Salazar et al (2016). Attrition analysis revealed that 1-month assessments were more likely to be completed by participants who were older (*t*_721.92_= 3.67; *P*<.001), not single (χ^2^_3_=11.467; *P*=.009), who used less alcohol in the 7 days prior to baseline (*t*_786.919_=–2.676; *P*=.008), who had higher sleep quality values (*t*_703.512_=–1.953; *P*=.05), who had more blog shares (*t*_482.08_=3.744; *P*<.001), blog comments (*t*_550.511_=4.058; *P*<.001), experiments taken (χ^2^_1_=15.65; *P*<.001), and finally, who stayed longer in the program (*t*_692.773_=5.252; *P*<.001). In addition, the 3-month assessments were also more likely completed by participants who were female (χ^2^_2_=20.366; *P*<.001), who had minimal initial suicide risk (χ^2^_2_=8.706; *P*=.01), and who had lower psychological distress (*t*_657.61_=–2.328; *P*=.02).

In the first round of analysis, WGEE models included only the time variable to examine significant changes in outcomes over the study course. In the second round, WGEE models included the following predictors: (1) time (baseline versus follow-up assessment); (2) study group; and (3) the interaction term for time by group. In the last round of analyses, WGEE models included: (1) time; (2) baseline variables; (3) program use variables; and (4) interaction terms for time by program use variables. We then used a hierarchical, backwards procedure where we removed predictors with the highest *P*-value one at a time until only significant predictors were retained within the model. In the WGEE parameters, the intercept shows the mean change in the outcome for all participants (and on a population level). Betas are added or subtracted to the intercept, which reveals the change in the outcome for the particular group.

Outcomes by AUDIT risk group (probably dependent versus hazardous or harmful) were assessed with a standard, repeated measures ANOVA, as we were interested in the subject level outcomes rather than the population interpretation from the WGEE. In addition, an alpha level of 0.05 (two tailed) was chosen for all statistical tests conducted in the study. All analyses were performed using the statistical tools SPSS version 22 and R version 3.3 via geepack [44].

## Results

### Study Participation, Sample Characteristics, and Attrition

**Figure 1:**
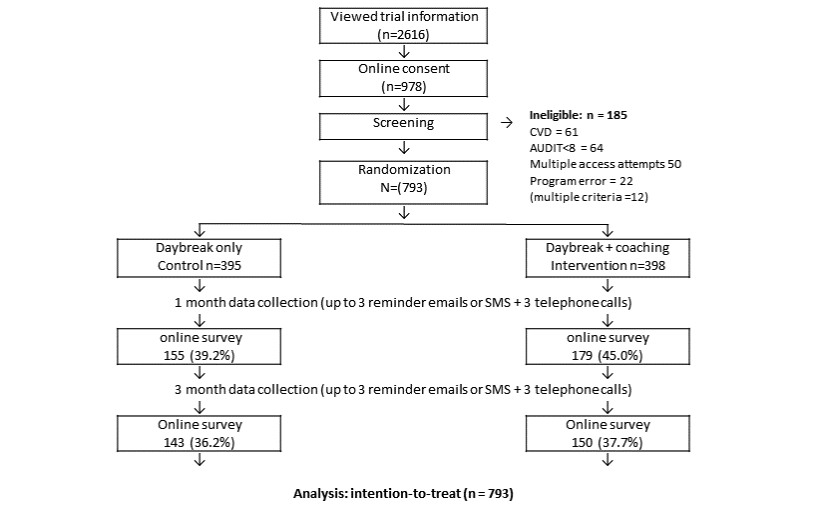
Consolidated standards of reporting trials diagram. CVD: cardiovascular disease. AUDIT: alcohol use disorders identification test.

There were 2616 people who viewed the invitation page. Of these, 1278 (49%) accessed additional information and 978 (77%) provided consent. However, not all were eligible for the study. Of everyone who provided consent, 61 indicated a previous cardiovascular disease diagnosis, 28 scored below the AUDIT cut-off score, and 36 did not complete the AUDIT (12 cases failed more than 1 eligibility criteria). Further, there were 50 people who attempted to enroll multiple times after failing an eligibility criterion. We excluded these people, plus an additional 22 people who were not randomized because of a programming error. Thus, the study cohort consisted of 793 people (81.1% of those who consented; see Figure 1).

There were 398 people randomized to the Intervention group (50.2%) and 395 to the Control group (49.8%). Of all the participants, 71% were female (one person selected other) with a mean age of 40.1 years (SD 10.0), and most participants (77%) had at least commenced tertiary education. The mean Kessler’s K-10 score was 24.8 (SD 7.0), with 19 participants (2.4%) reporting a serious suicide attempt, and nearly 30% of participants were in the highest risk category on the P4 suicide screen. A total of 69% (n=550) of participants were classified in the AUDIT highest risk category (probably dependent), with their mean alcohol consumption being 37.1 (SD 28.3) standard drinks per week. At baseline, the only significant difference between the groups was in terms of days completely out of role due to alcohol use (Table 1). Follow-up data were subsequently collected from 334 (42.1%) participants at 1 month, and from 293 (36.9%) at 3 months.

### Program Use

Overall, 106 participants engaged with coaching (eg, at least one message sent by the participant to the coach), including 68 from the Intervention and 38 from the Control groups (Table 2). A greater proportion of those in the Intervention group engaged with an online coach (17.1%; χ^2^_1_=9.5; *P*=.002) than the Control group (9.6%). Blog posts were made by 526 people, with 258 in the Intervention group (65%) and 268 in the Control group (68%). In terms of shares, 230 people (58%) in the Intervention and 227 people (58%) in the Control group commented on the posts of others. There were no significant differences in the median number of experiments taken, shares, comments or days in the study.

### Program Effectiveness

Pre-post comparisons of primary and secondary outcomes are displayed in Table 3. The WGEE models revealed significant changes over the study period for all outcomes at *P*<.001, except for amount of exercise at baseline to 1 month (*P*=.15) and at baseline to 3 months (*P*=.65). Participants significantly reduced their alcohol use and their psychological distress, and also gained in sleep quality and quality of life. Further, WGEE revealed that neither study group, nor the interaction term between study group and time, were associated with changes in primary or secondary outcomes. WGEE accounted for missing patterns at T1 and T2, with weights for T1 accounting for age, marital status, alcohol use days (last 7 days), sleep quality, retention, blog shares, blog comments, and experiments seen or done, and weights for T2 accounting for age, gender, marital status, suicide risk, psychological distress (K-10), retention, blog shares, blog comments, and experiments seen or done. Effect size was calculated via Psychometrica [45].

**Table 1:** Baseline characteristics (N=793).

Variable		Intervention (n=398)	Control (n=395)	*P* value
Sex (female)^a^, n (%)		285 (72)	276 (70)	.51
Age (years), mean (SD)		40.9 (10.0)	41.0 (10.1)	.89
**Marital status^a^, n (%)**				**.74**
	Single or divorced	139 (35)	129 (33)	
	Married	162 (41)	174 (44)	
	De facto	87 (22)	80 (20)	
	Remarried	10 (3)	12 (3)	
**Highest education level^a^, n (%)**				**.71**
	Primary or high school or trade	88 (22)	97 (25)	
	Some or complete university	191 (48)	185 (47)	
	Some or complete higher degree	119 (30)	113 (29)	
Suicide risk (highest category)^a^, n (%)		119 (30)	109 (28)	.30
Kessler-10, mean (SD)		24.5 (7.0)	25.0 (7.1)	.32
**Kessler-10 category^a^, n (%)**				**.62**
	Low	35 (9)	36 (9)	
	Mild	106 (27)	91 (23)	
	Moderate	154 (39)	168 (43)	
	Severe	103 (26)	100 (25)	
EUROHIS-QOL, mean (SD)		3.2 (0.7)	3.1 (0.7)	.70
Sleep Quality, mean (SD)		1.7 (0.7)	1.7 (0.8)	.70
Exercise (METS^b^)^c^, median (IQR)		36 (17-56)	35 (16-56)	.76
Health service use^c^, median (IQR)		2 (0-6)	2 (0-5)	.60
Core adverse events, mean (SD)		27.5 (11.3)	26.5 (12.1)	.24
**Days out of role^c^**				**.02**
	Median (IQR)	1 (0-2)	0 (0-1)	
	Mean (SD)	1.8 (4.1)	1.4 (2.8)	
**Part days out of role^c^**				**.47**
	Median (IQR)	1 (0-5)	1 (0-4)	
	Mean (SD)	3.6 (5.5)	3.5 (5.6)	
AUDIT^d^ (10 items; initial screen), mean (SD)		23.0 (6.0)	23.0 (6.5)	.61
**AUDIT 10 category^a^**				**.45**
	8-19 hazardous or harmful	117 (29)	126 (32)	
	20-40 dependent	281 (71)	269 (69)	
AUDIT–C (3 items), mean (SD)		9.0 (1.9)	9.2 (1.9)	.29
7-day standard drinks, mean (SD)		37.5 (31.1)	36.8 (25.3)	.74

^a^Assessed with chi-square test.

^b^METS: metabolic equivalents.

^c^Assessed with non-parametric Mann-Whitney *U*.

^d^AUDIT: alcohol use disorders identification test.

**Table 2:** Program use by study group (N=793).

Variable		Intervention (n=398)		Control (n=395)		*P* value	
Engaged with coaching (yes), n (%)		68 (17)		38 (10)		.002	
**Experiments taken (any)**							
	n (%)		147 (37)		134 (34)		.40
	Median (IQR^a^)		0 (0-1)		0 (0-1)		.50
Blog shares, median (IQR)		1 (0-4)		1 (0-10)		.13	
Blog comment, median (IQR)		1 (0-10)		2 (0-10)		.74	
Time in study (days), median (IQR)		32 (6-87)		30 (4-90)		.51	

^a^IQR: interquartile range.

**Table 3:** Means and standard deviations for outcomes, with significance tests of changes from T0 to T1 and T1 to T2, plus effect size (T0-T2). Please note: not all participants responded to all items in the survey, resulting in missing values.

Variable	Baseline (T0) (n=792)	1 month (T1) (n=333)	3 months (T2) (n=293)	*P* value (T0-T1)	*P* value (T0-T2)	Pre-post effect size (T0-T2)
AUDIT–C^a^, mean (SD)	9.11 (1.92)	6.03 (3.02)	5.78 (3.02)	<.001	<.001	–1.53
Alcohol days out of role, mean (SD)	1.59 (3.55)^b^	0.60 (2.46)^b^	0.48 (1.63)	<.001	<.001	–0.262
Alcohol use (last 7 days), mean (SD)	37.10 (28.34)^c^	17.06 (21.57)	17.49 (18.89)	<.001	<.001	–0.689
Kessler K-10, mean (SD)	24.80 (7.03)	19.84 (6.75)	18.97 (6.60)	<.001	<.001	–0.937
EUROHIS, mean (SD)	3.15 (0.70)	3.51 (0.73)	3.57 (0.70)	<.001	<.001	0.745
Sleep Quality, mean (SD)	1.70 (0.74)^d^	1.28 (0.74)	1.27 (0.75)	<.001	<.001	–0.54
Exercise (METS^e^), mean (SD)	55.41 (153.19)	76.20 (246.16)	52.05 (97.88)	.15	.65	–0.015

^a^AUDIT–C: alcohol use disorders identification test-communication.

^b^11 missing values.

^c^1 missing value.

^d^4 missing values.

^e^METS: metabolic equivalents.

Change in alcohol consumption was compared for those who were as classified on the AUDIT–10 at baseline as hazardous or harmful or as probably dependent. Retention was similar for the two groups at three months, with hazardous or harmful at 38.3% (χ^2^_1_=0.3; *P*=.61) versus probably dependent at 36.4 % (χ^2^_1_=0.3; *P*=.61). There were significant reductions in alcohol use for both groups (*F*_1288_*=* 24.7; *P*<.001), with estimated marginal means of 40.8 down to 20.1 drinks for probably dependent and 22.9 down to 11.9 drinks for hazardous or harmful, and there was also a significant time by group interaction (*F*_1288_*=* 8.3; *P*=.004) reduction for the probable dependence group. The time by sex and the time by group by sex interactions were not significant.

Factors that predicted significant changes in alcohol-related variables are displayed in Table 4. Online coaching was not significant in any of the models and was therefore not retained. Significant predictors for AUDIT–C were time (reduced at each follow-up), gender (males report higher scores than females), age (older participants report higher scores than younger participants), and the interaction between blog comments and time (more comments were associated with lower AUDIT–C scores at 1-month follow-up). Significant predictors for alcohol days out of role were time (reduced at each follow-up), gender (males report more days out of role than females), marital status (single participants report more days out of role than partnered participants), education (higher education was associated with lower scores), and the interaction between taking part in experiments and time (taking part in experiments was associated with fewer days out of role at 1-month and 3-months follow-up). Significant predictors for alcohol use in standard drinks were time (lower at each follow-up), age (increased with age), marital status (lower for partnered people), and the interaction term between blog shares and time (more shares were associated with lower alcohol use at 1-month and 3-months follow-up).

**Table 4:** Factors relating to change in alcohol use disorders identification test (AUDIT–C), alcohol days out of role and alcohol use (last 7 days) for all participants (Weighted Generalized Estimating Equation models).

Model, predictors, categories			Beta	SE	*P* value
**AUDIT–C**					
	Model intercept		8.03	0.4	<.001
	**Time**				
		Baseline (ref^a^)	—^b^	—	—
		1 month	–2.88	0.17	<.001
		3 months	–3.03	0.21	<.001
	**Gender**				
		Female (ref)	—	—	—
		Male	0.76	0.23	.001
	Age		0.02	0.009	.04
	Blog comments		0.003	0.0009	<.001
	**Blog comments × time**				
		Baseline (ref)	—	—	—
		1 month	–0.008	0.003	.003
		3 months	–0.004	0.003	.13
**Alcohol days out of role**					
	Model intercept		2.03	0.21	<.001
	**Time**				
		Baseline (ref)	—	—	—
		1 month	–0.58	0.27	.03
		3 months	–0.94	0.17	<.001
	**Gender**				
		Female (ref)	—	—	—
		Male	0.45	0.22	.04
	**Marital status**				
		Single or divorced (ref)	—	—	—
		Married or remarried or de facto	–0.83	0.19	<.001
	**Education level**				
		Lower (ref)	—	—	—
		Higher	–0.46	0.16	.004
	**Experiments**				
		No (ref)	—	—	—
		Yes	0.32	0.27	.23
	**Experiments × time**				
		Experiments (no) × baseline (ref)	—	—	—
		Experiments (yes) × 1 month	–0.86	0.36	.01
		Experiments (yes) × 3 months	–0.25	0.3	.40
**Alcohol use last 7 days**					
	Model intercept		26.15	3.19	<.001
	**Time**				
		Baseline (ref)	—	—	—
		1 month	–19.17	1.46	<.001
		3 months	–18.06	1.42	<.001
	Age		0.26	0.08	.002
	**Marital status**				
		Single or divorced (ref)	—	—	—
		Married or remarried or de facto	–3.93	1.97	.04
	Blog shares		0.05	0.05	.26
	**Blog shares × time**				
		Baseline (ref)	—	—	—
		1 month	–0.13	0.06	.04
		3 months	–0.14	0.05	.01

^a^Reference category.

^b^Not applicable.

Factors that significantly predicted changes in psychological distress and quality of life are displayed in Table 5. Significant predictors for the K-10 were time (lower scores at both follow-ups), age (lower with increasing age), marital status (lower for partnered participants), and education level (lower for those with more education). None of the interaction terms for program use by time were retained in the model. Significant predictors for EUROHIS-QOL were time (higher scores at each follow-up), marital status (higher for partnered participants), education (higher for those with more education), and the interaction between blog comments and time (more comments were associated with higher quality of life scores at 1-month follow-up).

**Table 5:** Factors relating to changes in K-10 and EUROHIS-QOL scores for all participants (WGEE models).

Model, predictor, and categories			Beta	SE	*P* value
**Kessler K-10**					
	Model intercept		28.76	1.14	<.001
	**Time**				
		Baseline (ref^a^)	—^b^	—	—
		1 month	–4.77	0.36	<.001
		3 months	–5.33	0.42	<.001
	Age		–0.06	0.03	.04
	**Marital status**				
		Single/divorced (ref)	—	—	—
		Married/remarried/de-facto	–1.62	0.57	.005
	**Education level**				
		Lower (ref)	—	—	—
		Higher	–1.86	0.56	<.001
**EUROHIS-QOL**					
	Model intercept		2.9	0.04	<.001
	**Time**				
		Baseline (ref)	—	—	—
		1 month	0.32	0.03	<.001
		3 months	0.39	0.04	<.001
	**Marital status**				
		Single/divorced (ref)	—	—	—
		Married/remarried/de-facto	0.3	0.05	<.001
	**Education level**				
		Lower (ref)	—	—	—
		Higher	0.17	0.05	.001
	Blog comments		–0.0007	0.003	.03
	**Interaction blog comments** **×** **time**				
		Baseline (ref)	—	—	—
		1 month	0.001	0.0005	.03
		3 months	0.0007	0.0005	.17

^a^Reference category.

^b^Not applicable.

## Discussion

### Principal Findings

Use of the *Daybreak* program resulted in significantly improved outcomes in terms of alcohol measures, mental health and quality of life. An important finding was that the program was effective in those who were classified as probably dependent at baseline. Face to face brief interventions are recommended for those with at-risk alcohol use, but they are regarded as ineffective for those with alcohol use disorders [46,47]. It is therefore important to determine the range of alcohol use for which electronic health (eHealth) interventions are effective. Overall, these findings need to be tempered with the fact that the trial was not implemented as designed, and the longest period of follow-up was only three months.

With its multiple components, the *Daybreak* program does not fit within the typical definitions of a brief intervention, certainly not as described for face to face brief interventions [46,48]. In the context of eHealth interventions, the concept of a number of sessions of treatment or time to deliver treatment is ambiguous; however, Riper et al note a distinction is often drawn between “single session e-personalized normative feedback” and extended interventions that draw on a range of therapeutic techniques [11]. The *Daybreak* program lies within the more extensive group. Previously, the extent of engagement with the *Hello Sunday Morning* program (eg, in terms of blog posts, blog shares, following other participants) has been shown to correlate with improved outcomes (ie, reduced AUDIT scores) [19]. Despite the low overall level of engagement (see Table 2), these measures still predicted a range of improved outcomes (eg, in AUDIT–C score, in EUROHIS-QOL score with blog comments about alcohol use, and in blog shares at one month). These findings suggest that the community aspect of *Daybreak* has an important therapeutic role in supporting behavior change.

The outcomes for the study also need to be considered in light of the cohort who participated. Those who access eHealth interventions often have less extensive alcohol use, with a range of 9.1-43.6 standard drinks (mean 18.3) [12], than those in face to face interventions (mean 24.4 standard (10g) drinks) [49]. However, in the recent analysis by Riper et al, the mean consumption was 38.1 standard drinks per week [13]. Most of our participants were classified as probably dependent on alcohol, with mean consumption being 37.1 standard drinks, but the prevalence of severe alcohol problems was higher than typically found in eHealth trials. Thus, many of our participants (69%) were classified as probably dependent on the AUDIT whereas the respective figure reported by Riper et al was 22%, but they also reported that 34% were heavy drinkers (defined as >35/50 standard drinks for females or males) [13]. Also notable was the high prevalence of women in our trial, representing 71% of participants compared with typical values of about 50% [12,13] and even compared to the previous assessment of *Hello Sunday Morning* clients (64%) [19]. There are concerns that eHealth alcohol interventions may not be as effective with women as men [13]. Although men had higher AUDIT–C scores and more days out of role than women, gender was not significant as an interaction term in the current study.

Also notable was the high level of distress as measured with the K-10 and the suicide screening scores. Given the evident distress, it is important that eHealth interventions in this area provide appropriate support and referral pathways. In contrast, the days out of role were not as extensive as those reported for people with alcohol dependence [32], and the mean quality of life scores, while lower at baseline than the general population, were above those for a cohort with depression [34]. Further, by three months the mean score was above that for a general population sample [32].

One of the main aims of this trial was to evaluate if outcomes could be enhanced by giving participants access to online coaching. Few participants opted to use this facility, with 17.1% of the Intervention group accessing the service. Overall, online coaching was not associated with any significantly improved outcomes. The lack of an effect for online coaching is similar to previous studies where guidance has not improved alcohol-related outcomes [24], but remains in contrast to both eHealth interventions for other mental health problems where guidance appears to be beneficial (eg, standardized mean difference compared to unguided=–0.27) [50] and the most recent analysis of alcohol interventions [13]. The reason for the potential difference between those with alcohol use problems and other types of mental health issues is unclear but given that improved outcomes were obtained without the provision of this additional resource-intensive component, this means that *Daybreak* can be provided more widely.

### Limitations

Clearly the most significant limitation was that the trial was not implemented as designed, with some members of the Control group accessing the online coaching, so this necessitated a change in the planned analysis. In addition, we also stopped the trial at the completion of the three-month follow-up rather than at six months due to this error. Compared with other studies in the field, the level of attrition for this study was high (63%) compared to other alcohol studies of at least three months duration (range=8%-45%; mean 25.2% [12]) [24], thus limiting the generalizations that can be made from these data.

### Conclusions

The current Australian guidelines do not provide a formal recommendation for the number of drinks per week that constitute low risk drinking, but they do recommend that average daily consumption should not exceed two drinks per day [3], equating to 14 per week. On this basis, the average reported here (17.5 drinks) still exceeds that figure, albeit from a high starting point. We do note that those with hazardous or harmful alcohol use did reduce their consumption to below the guideline (mean 11.9 at three months). The *Daybreak* program, due to its extensive reach, member engagement and clinical safety, has the potential to realize a population level impact for both at risk drinkers and for probably dependent drinkers [15].

## Appendix

Multimedia Appendix 1 Participant information.

Multimedia Appendix 2 Outcomes by randomized groups.

Multimedia Appendix 3 CONSORT-eHealth checklist (V1.6).

## Abbreviations

ANOVA: analysis of variance
AUDIT: alcohol use disorders identification test
eHealth: electronic health
MCAR: missing completely at random
WGEE: Weighted Generalized Estimating Equation
