# Supplementary material for: A Digital Intervention Addressing Alcohol Use Problems (the “Daybreak” Program): Quasi-Experimental Randomized Controlled Trial
Source: J Med Internet Res. 2019 Sep 4;21(9):e14967. doi: 10.2196/14967 (PMC6753690; doi:10.2196/14967)
Supplement: Multimedia Appendix 1 [file jmir_v21i9e14967_app1.pdf]

national drug research institute

National Drug Research Institute
Curtin University
Health Research Campus
GPO Box U1987 Perth WA 6845
*telephone* +61 8 9266 1600
*facsimile* +61 8 9266 1611
ndri@curtin.edu.au
ndri.curtin.edu.au
*Preventing harmful drug use in Australia*
*Funded by the National Drug Strategy*
CRICOS Provider Code 00301J (WA), 02637B (NSW)

# Information sheet
# Evaluating the effectiveness of the Hello Sunday Morning *Daybreak* program in reducing alcohol use

Hello Sunday Morning has asked the National Drug Research Institute to test how well the *Daybreak* program works. We are testing different versions of the program and support services to find out which one is the most effective. If you agree to take part in the study, you will be allocated at random to one of two versions.

## Who can take part?

We are looking for people aged 18 or older, who currently drink alcohol in a manner that puts their health 'at risk', for example who have incurred an injury, done something that they later regret or who simply want to reduce their alcohol consumption. In addition, you must be currently living in Australia.

## What does the research involve?

If you agree to take part in the study, first, we'll collect some basic information about your use of alcohol and your health to make sure that the study is suitable for you. We will also need a phone number and contact e-mail address. We'll ask you to create your own username and password so that your answers at each stage of the study can be combined. We will ask you to complete some detailed questions on your alcohol use, mental health, wellbeing, exercise, quality of life and any adverse events related to alcohol use. You will then be randomised to one of two versions of Daybreak. We will contact you at 1 and 3 months to find out how you are progressing. Each survey should take less than 30 minutes.

## What does the program offer?

The content of the program will vary depending on which group you are randomised to. However, there are common element. *Daybreak* encourages members to take "experiments", and reflect on their learnings, for example in recognising and dealing with "drinking triggers". All participants have access to the supportive *Daybreak* online peer community and are also encouraged to reflect on what motivates them to drink.

## Do I have to take part in the study?

No, the study is completely voluntary; you are free to withdraw at any time without prejudice or negative consequences. If you decide not to take part, or if you later withdraw, it will have no impact your future access to the Hello Sunday Morning web-site and services.

## Are the results confidential?

Your personal information will remain confidential: Hello Sunday Morning will hold any information that you supply securely. A copy of your surveys, information you post on *Daybreak* and details of how often you visit it will be provided by Hello Sunday Morning to the National Drug Research Institute (NDRI), which is part of Curtin University, together with an ID number rather than your personal details. The researchers will not be able to identify you unless you include identifying details when you post information (for example, as part of your shares or comments). Even if you

did include personal information, any publications about the study will not include any identifying information about you or other participants.

**Are there any risks in taking part?**

The risk of taking part in the study is the same or lower than for someone using the *Daybreak* program who doesn't take part in the study. We suggest you use a nick name and do not reveal your contact details on the app to make sure your privacy is completely secure.

**Is there any benefit to taking part?**

To acknowledge your help with the research, you will have 12 months of free access to the version of the *Daybreak* service to which you are randomised. In addition, we are offering entry into a prize draw at each follow-up survey (at 1 and 3 months) for an iPad 2.

**What treatment services will be available at the end of the study?**

Hello Sunday Morning is committed to providing the best evidence-based services available. Outcomes from the study will be used to try to obtain funding for future improved services. However, the availability and form of services after the end of the study are dependent on the extent of funding obtained.

**Whom should I contact if I have further questions or complaints about the study?**

For further information contact Dr Robert Tait (08 92661610 or e-mail robert.tait@curtin.edu.au).

**What should I do next?**

If you would like to take part in the study click here

If you do not want to take part in the study click here

*This study has been approved by the Curtin University Human Research Ethics Committee (Approval Number HRE2017-0855). The Committee is comprised of members of the public, academics, lawyers, doctors and pastoral carers. If needed, verification of approval can be obtained either by writing to the Curtin University Human Research Ethics Committee, c/- Office of Research and Development, Curtin University, GPO Box U1987, Perth, 6845 or by telephoning 9266 9223 or by emailing hrec@curtin.edu.au*
