# Supplementary material for: A Digital Intervention Addressing Alcohol Use Problems (the “Daybreak” Program): Quasi-Experimental Randomized Controlled Trial
Source: J Med Internet Res. 2019 Sep 4;21(9):e14967. doi: 10.2196/14967 (PMC6753690; doi:10.2196/14967)
Supplement: Multimedia Appendix 2 [file jmir_v21i9e14967_app2.pdf]

Supplementary file 2: Outcomes by randomized groups

Variable	Intervention			Control			All participants		
	Baseline n= 398	1 month n=179	3 month n=150	Baseline n= 395	1 month n=155	3 month n=143	Baseline n=793	1 month n=334	3 month n=293
AUDIT-C *M (SD)*	9.04 (1.89)	6.20 (3.02)	5.78 (3.01)	9.19 (1.94)	5.82 (3.02)	5.78 (3.14)	9.11 (1.92)	6.02 (3.02)	5.78 (3.07)
Alcohol days out of role *M (SD)* *Median (IQR)*	1.82 (4.12) [a] 1 (0-30)[a]	0.84 (3.20) 0 (0-30)	0.67 (2.14) 0 (0-15)	1.35 (2.84) [b] 0 (0-20)[b]	0.32 (1.04)[c] 0 (0-10) c	0.27 (0.76) 0 (0-4)	1.59 (3.55)[d] 0 (0-30) [d]	0.60 (2.46)[c] 0 (0-30) [c]	0.48 (1.63) 0 (0-15)
Alcohol use, last 7 days *M (SD)*	37.45 (31.09)	17.54 (19.87)	18.22 (18.18)	36.77 (25.27) c	16.39 (23.41)	16.73 (19.64)	37.12 (28.33)c	17.01 (21.56)	17.49 (18.89)
Kessler-10 *M (SD)*	24.54 (6.99)	19.40 (6.75)	18.53 (6.59)	25.04 (7.07)	20.32 (6.72)	19.44 (6.61)	24.79 (7.03)	19.82 (6.74)	18.97 (6.61)
EUROHIS-QoL *M (SD)*	3.16 (0.70)	3.52 (0.69)	3.56 (0.70)	3.14 (0.71)	3.49 (0.76)	3.58 (0.70)	3.15 (0.70)	3.51 (0.73)	3.57 (0.70)
Sleep Quality *M (SD)*	1.71 (0.71) c	1.32 (0.73)	1.32 (0.77)	1.69 (0.76) f	1.21 (0.76)	1.21 (0.73)	1.70 (0.74)e	1.27 (0.74)	1.27 (0.75)
Exercise *M (SD)*	59.32 (183.43)	63.14 (174.22)	45.37 (36.18)	51.34 (114.83)	90.89 (308.48)	59.05 (135.02)	55.34 (153.10)	76.02 (245.81)	52.05 (97.88)
Health service use last 8 weeks *M (SD)*	4.33 (5.49)	4.42 (12.52)	3.66 (4.44)	4.00 (5.29)	3.54 (4.75)	3.08 (4.05)	4.17 (5.39)	4.01 (9.71)	3.38 (4.26)

IQR = Interquartile range: M = mean: Exercise in Metabolic equivalents (METS) : SD = standard deviation

Missing values: a: 5, b: 6, c: 1, d: 11, e: 4, f: 3
